# Prevalence and effects of Vitamin D receptor polymorphism on bone mineral density and metabolism in patients with systemic sclerosis: a preliminary study

**DOI:** 10.1007/s10238-024-01385-1

**Published:** 2024-06-07

**Authors:** Nils Schulz, Gabriel Dischereit, Laura Henke, Uwe Lange, Philipp Klemm

**Affiliations:** Department of Rheumatology, Clinical Immunology, Osteology and Physical Medicine, Justus-Liebig-University Giessen, Campus Kerckhoff, Benekestr. 2-8, 61231 Bad Nauheim, Germany

**Keywords:** Systemic sclerosis, Bone mineral density, VDR polymorphisms

## Abstract

Patients with systemic sclerosis (SSc) have a disproportionately high prevalence of reduced bone mineral density (BMD). Polymorphisms of the vitamin D receptor (VDR) gene have been associated with osteoporosis in patients with autoimmune diseases. The aim of this study was to investigate the prevalence and possible effects of VDR polymorphism on BMD and bone metabolism in patients with SSc. In patients with SSc measurement of BMD was performed using dual-energy X-ray absorptiometry. VDR polymorphisms (FokI, BsmI) were genotyped using restriction fragment length polymorphism analysis. Markers of bone metabolism (calcium, osteocalcin, β-crosslaps) were determined. Primary endpoint was the prevalence of VDR gene polymorphisms and the association with reduced BMD. Secondary endpoints included associations between bone metabolism and VDR gene polymorphism. 79 Caucasian patients with SSc were included. Overall, 83.5% had reduced BMD (51.9% osteopenia, 31.6% osteoporosis). The prevalence of VDR gene polymorphism (73% BsmI, 77% FokI) was comparable to studies in healthy and rheumatic populations. The homozygous presence of FokI polymorphism, but not BsmI, was significantly associated with reduced axial BMD. Fokl polymorphism was significantly associated with reduced CTX levels, although changes remained within the reference limits. VDR polymorphisms can frequently be found in patients with SSc in comparable prevalence to healthy and rheumatic populations. The homozygous presence of FokI polymorphism, but not BsmI, was significantly associated with reduced axial BMD. This could be a possible contributor for the high prevalence of reduced BMD in 83.5% of patients with SSc in this study.

*Trial registration*. DRKS00032768, date: 05.10.2023, retrospectively registered.

## Introduction

Systemic sclerosis (SSc) is a rare autoimmune connective-tissue disease with a prevalence of approximately 1:5.000 [1, 2]. Hallmarks of this complex disease are fibrosis of the skin and internal organs, vasculopathy and autoimmunity [3]. Although it is a rare disease, the disease burden is substantial. SSc has this highest mortality of all rheumatic disease [1]. Moreover, SSc is disproportionately highly associated with reduced bone mineral density (BMD) and osteoporosis. Osteoporosis is a systemic skeletal disorder characterized by low bone mass and microarchitectural deterioration of the bone tissue, resulting in increased bone fragility and susceptibility to fractures [4]. Osteoporosis is considered the most common metabolic bone disease in elderly population and will continue to gain significance due to demographic changes [5]. Presently, osteoporosis is acknowledged as one of the economically most impactful diseases of the twenty-first century, accounting for a total healthcare expenditure of approximately 20% of the respective gross domestic product [6]. Furthermore, osteoporosis-related fractures are associated with increased mortality, with a 20–25% excess mortality rate within the first 6 months after a hip-proximal fracture [7, 8]. Therefore, the combination of SSc and osteoporosis has a highly negative impact for the individual.

There is a strong evidence that patients with SSc have a heightened susceptibility to osteoporosis: A systematic review covering the years from 1948 to 2012 identified 15 studies comparing the BMD of SSc patients with healthy control groups showing that patients with SSc have an elevated risk of reduced BMD and fractures, especially in the presence of other risk factors for osteoporosis [9]. A meta-analysis based on data of 662 SSc patients and 886 controls confirmed: BMD is lower in SSc patients than in healthy controls, both in the lumbar spine and the femoral neck [10]. Both forms of SSc, limited cutaneous (lc) and diffuse cutaneous (dc) SSc, exhibited the same risk.

Although osteoporosis is a major health problem, the multifactorial pathogenesis of osteoporosis is poorly studied, especially in the context of SSc. Hereditary factors seem to play an important role [11, 12]. In particular, associations between polymorphisms of the vitamin D receptor (VDR) gene and low BMD have been demonstrated [13]. The VDR has high affinity for calcitriol and is responsible for its hormonal effects as a ligand-activated transcription factor [14]. Based on single nucleotide polymorphisms more than 900 allelic variants of the VDR gene are known, that can result in lower binding qualities of the receptor for calcitriol, which subsequently leads to alterations in vitamin D and calcium metabolism [14, 15]. More importantly, specific length polymorphisms of restriction fragments could be found that influence BMD, particularly in patients with inflammatory rheumatic joint diseases [16]. The restriction enzymes BsmI (rs1544410), TaqI (rs731236), and ApaI (rs7975232) cleave polymorphic regions located in the intron at the 3’-end of the VDR gene, with the BsmI and ApaI polymorphisms located in intron 9 and TaqI in exon 9. Another polymorphism located in exon 2 at the 5’-end of the gene can be detected using the FokI restriction enzyme (rs2228570) [17, 18].

Prevalence of VDR gene variants FokI and BsmI varies and is partly dependent on the studied population, suggesting an ethnic background. Variant alleles of VDR BsmI and FokI were found in 50% and, respectively, 25% of healthy Saudi Arabian probands [19]. However, in a Lithuanian study on VDR polymorphisms and rheumatoid arthritis (RA), FokI polymorphisms were found in 76.1% of patients with RA and 78.9% of the control group, while BsmI polymorphisms were detected in 55.8% of patients with RA and 55.6% of the control group [20].

Within the context of BMD and inflammatory rheumatic joint diseases, female patients with RA who were homozygous carriers of the TaqI restriction endonuclease cleavage sites (TT genotype) exhibited a more rapid loss of bone mass compared to those without the genotype (tt genotype) [21]. In an Italian population, the distribution of VDR gene polymorphisms did not differ between patients with RA and healthy subjects (FokI: 92% vs 90%, TaqI: 85% vs 88%, BsmI: 55% vs 58%, and ApaI: 98% vs 98%). Similar to the work of Gough et al., the VDR gene polymorphism TaqI showed a statistically significant association with decreased axial and peripheral BMD, but only in female patients and not in males [22]. In male patients with ankylosing spondylitis (AS), the presence of the FokI VDR polymorphism genotype was significantly associated with lower BMD at the lumbar spine, but not at the femoral neck. The presence of the FokI genotype was identified as an independent predictor of lower BMD values, overall FokI VDR polymorphism could be detected in 85% of probands [16].

Therefore, this study investigated the prevalence of VDR gene polymorphisms in patients with SSc and whether they are associated with changes in BMD and bone metabolism.

## Methods

All patients treated at our center with diagnosed SSc fulfilling the ACR/EULAR classification criteria of 2013 were eligible for this study [23]. Patients were excluded if they had a medical history of secondary osteoporosis or if baseline work-up revealed such. Patients receiving glucocorticoid therapy were excluded. Recruitment and data collection were conducted at Justus Liebig University Giessen, Campus Kerckhoff in Bad Nauheim. All patients were thoroughly informed about the study and provided written consent to participate. The study was approved by the Ethics Committee of Justus Liebig University Giessen (AZ 85/12). The study was registered in the German registry of clinical studies (DRKS) with the number DRKS00032768.

VDR gene polymorphisms were determined from DNA using restriction enzymes FokI and BsmI. DNA was initially extracted from peripheral leukocytes (whole blood samples) using the QIAamp DNA Blood Kit (Quiagen Ltd, Max-Volumer-Strasse, Hilden, Germany), amplified and separated by gel electrophoresis. The corresponding gene mutations were detected using restriction fragment length polymorphism (RFLP) analysis with the assistance of restriction endonucleases.

The absence of the BsmI endonuclease cleavage site (wildtype) was denoted as (B), while its presence was denoted as (b). Similarly, the absence of the FokI enzyme cleavage site (wildtype) was denoted as (F), and its presence was denoted as (f). This resulted in three variants for each polymorphism: (BB), (Bb), and (bb) for BsmI, and (FF), (Ff), and (ff) for FokI.

BMD measurements were performed using dual-energy X-ray absorptiometry (DXA) with a Lunar Prodigy X device (Lunar Radiation Corporation, 313 West Beltline Highway, Madison, Wisconsin 53,713, USA). Measurements were taken at the lumbar spine (L1-L4) and the entire right femoral neck. According to WHO criteria, a decrease in BMD of > 2.5 standard deviations below the mean value for young healthy adults (T-score < -2.5) was defined as osteoporosis. A T-score between -2.5 and -1 was classified as osteopenia, while a T-score > -1 indicated age-appropriate BMD [24].

Sensitive pre-analytical procedures included standardized blood collection in the early morning for serum calcium, 25-hydroxy vitamin D, C-reactive protein (CRP), erythrocyte sedimentation rate (ESR), as well as bone metabolism markers osteocalcin (OC) and β-crosslaps (CTX) in all patients. To further exclude potential confounding factors of bone metabolism (secondary forms of osteoporosis) such as thyroid or renal dysfunction, thyroid-stimulating hormone (TSH) and serum creatinine were also measured.

The primary endpoint of the study was the investigation of (preliminary) prevalence of VDR gene polymorphisms and the statistical association between VDR gene polymorphisms (FokI and/or BsmI genotypes) and decreased BMD/osteoporosis measured by DXA. Secondary endpoints included statistical associations between laboratory values, including bone metabolism parameters, and VDR gene polymorphisms.

Metrically scaled parameters are presented descriptively with arithmetic mean, standard deviation, and confidence interval for the mean. Data were examined for normal distribution using Q-Q plots and the Shapiro–Wilk test. For non-normally distributed data, the nonparametric U-test was used for comparisons. The nonparametric Kruskal–Wallis test was used for comparisons among three groups, followed by pairwise comparisons using the Dunn-Bonferroni test. Categorical parameters were analyzed for associations using Fisher’s exact test. The alpha level for the study was set at p = 0.05. The analysis was performed using the R program for Windows version 3.3.2. The analysis was conducted by assessors blinded to the study.

## Results

**Fig. 1:**
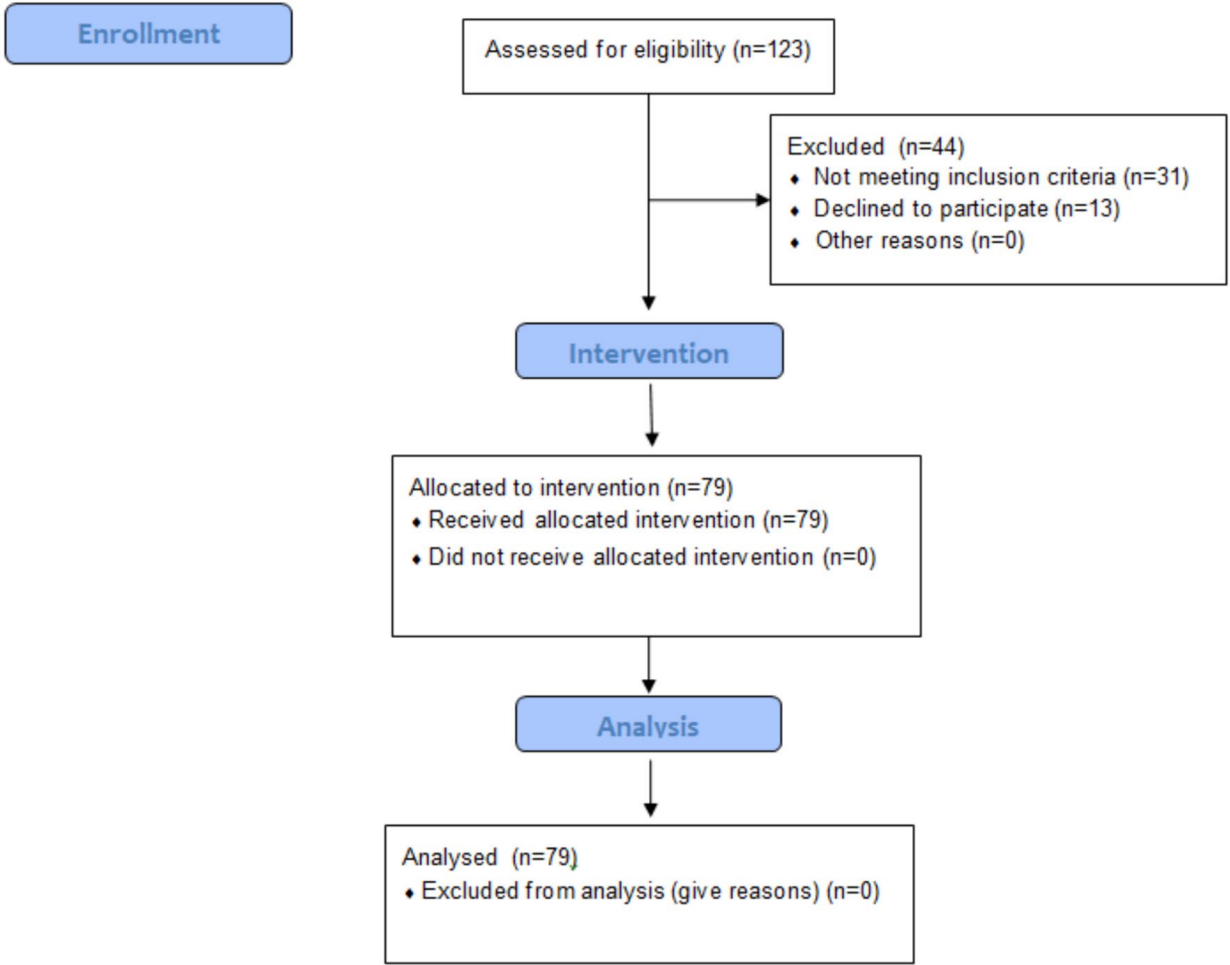
CONSORT 2010 Flowchart. In the enrollment, 123 patients were screened for inclusion and exclusion criteria. 44 were excluded: 31 due to secondary osteoporosis or glucocorticoid therapy. 13 patients did not want to participate in the study. The intervention (VDR gene polymorphism analysis) was conducted on 79 patients. All 79 patients were included in the analysis and contributed to the results

After screening our center’s SSc cohort of 123 patients a total of 79 Caucasian SSc patients (68 females, 11 males, mean age 66 years) met both inclusion and exclusion criteria and were willing to participate and thus included. Of the 44 patients not taking part in the study 31 were excluded due to secondary osteoporosis and/or glucocorticoid-treatment and 13 did not want to participate (cf. Fig. 1).

The differentiation between lc and dcSSc was based on Le Roy’s classification [25].

Further patient characteristics are shown in Table 1.

**Table 1:** Patient characteristics at inclusion

Characteristic	*n* (%)
Number of patients	79 (100%)
Gender	
Female	68 (87.3%)
Male	11 (12.7%)
Age in years (mean, SD)	66.3 ± 11.2
Disease duration in years (mean, SD)	14 ± 7.9
Form of SSc	
lcSSc	57 (72.2%)
dcSSc	22 (27.8%)
Antibody profile	
Anti-Scl-70	29 (36.7%)
Anti-centromere	50 (63.3%)
Organ manifestation	
Interstitial lung disease	39 (49.4%)
Pulmonary hypertension	24 (30.4%)
Digital ulcerations	37 (46.8%)
Esophageal atony	56 (70.9%)

*n* number, *lc/dcSSc* limited/ diffuse cutaneous systemic sclerosis

### BMD and osteospecific treatment

Of 79 included SSc patients, 25 (31.6%) had osteoporosis and 41 (51.9%) had osteopenia. Only 13 patients showed a normal BMD on DXA. In total, 83.5% (66/79) of the included patients with SSc had a reduced BMD. 28 patients with lcSSc and 13 patients with dcSSc had osteopenia, while 19 patients with lcSSc and 6 patients with dcSSc had osteoporosis (Table 2).

**Table 2:** BMD measured by DXA depending on the subtype of SSc

BMD	Total	lcSSc	dcSSc
Normal (T-score > -1)	13 (16.5%)	10 (17.5%)	3 (13.6%)
Osteopenia (T-score -1 to -2,5)	41 (51.9%)	28 (49.1%)	13 (59.1%)
Osteoporosis (T-score < -2,5)	25 (31.6%)	19 (33.3%)	6 (27.3%)
Total	79 (100%)	57 (100%)	22 (100%)

*lc/dcSSc* limited/diffuse cutaneous systemic sclerosis

The subtyp of SSc (lc/dcSSc) was not associated with osteopenia/osteoporosis (*p* = 0.789).

In the group of 25 osteoporosis patients, 22 patients met thresholds for treatment based on German recommendations and received osteospecific therapy [26]. No patient with normal BMD or osteopenia received osteospecific therapy.

All patients received antiresorptive osteospecific therapy either intravenously with zoledronate 5 mg once a year (*n* = 16) or subcutaneously with denosumab 60 mg every 6 months (*n* = 6). Due to high prevalence of esophageal atony and gastric reflux in patients with SSc, bisphosphonate treatment is administered intravenously in our center.

### VDR gene polymorphisms

VDR polymorphisms BsmI and FokI were both highly prevalent and could be found in the majority of patients. BsmI polymorphism was found in 58 of 79 (73%) patients and FokI polymorphism was found in 61 of 79 (77%) patients. 44 of 79 patients (55.7%) had both BsmI and FokI VDR polymorphisms, whereas 31 of 79 patients (39.2%) had only either FokI or BsmI VDR polymorphism. Only 4 of 79 patients (5.1%) had neither FokI nor BsmI VDR polymorphism. In the comparison of patients with both VDR gene polymorphisms (BsmI and FokI) to patients without VDR gene polymorphisms, there was no statistically significant difference in axial BMD (*p* = 0.547).

Twelve patients with lcSSc showed a homozygous presence of the BsmI restriction endonuclease site (bb), 30 patients had a heterozygous genotype (Bb), and 15 patients had a homozygous absence of the BsmI restriction endonuclease site (BB). Among patients with dcSSc, 6 had a homozygous genotype for the presence of the BsmI restriction endonuclease site (bb), 10 had a heterozygous type (Bb), and in 6 patients, the site was absent on both alleles (BB). BsmI was neither significantly associated with lc- or dcSSc (*p* = 0.804) nor with gender (*p* = 0.837).

Regarding the FokI genotype, 13 patients with lcSSc had a homozygous presence of the FokI restriction endonuclease site (ff), 26 patients had a heterozygous genotype (Ff), and 18 patients had a homozygous absence of the restriction enzyme site (FF). 5 patients with dcSSc had a homozygous presence of the FokI restriction endonuclease site (ff), while 11 patients had a heterozygous type (Ff), and 6 patients had a homozygous absence (FF). FokI was neither significantly associated with lc- or dcSSc (*p* = 0.947) nor with gender (*p* = 0.640).

There was no statistically significant correlation between the presence of individual VDR gen polymorphisms FokI and BsmI (*p* = 0.888).

Table 3 shows the frequency of BsmI/FokI genotypes in the different forms of SSc.

**Table 3:** Frequency of the BsmI/FokI genotypes in lc and dcSSc

	Total	lcSSc	dcSSc
BsmI			
bb	18 (22.8%)	12 (21.1%)	6 (27.3%)
Bb	40 (50.6%)	30 (53.6%)	10 (45.4%)
BB	21 (26.6%)	15 (26.3%)	6 (27.3%)
Total	79 (100%)	57 (100%)	22 (100%)
FokI			
ff	24 (30.1%)	18 (31.6%)	6 (27.3%)
Ff	37 (47.1%)	26 (45.6%)	11 (50%)
FF	18 (22.8%)	13 (22.8%)	5 (22.7%)
Total	79 (100%)	57 (100%)	22 (100%)

l*c/dcSSc* limited/diffuse cutaneous systemic sclerosis

Table 4 shows the frequency of BsmI/FokI genotypes in different gender.

**Table 4:** Frequency of the BsmI/FokI genotypes in gender

	Total	Female	Male
BsmI			
bb	18 (22.8%)	16 (23.5%)	2 (18.2%)
Bb	40 (50.6%)	34 (50%)	6 (54.5%)
BB	21 (26.6%)	18 (26.5%)	3 (27.3%)
Total	79 (100%)	68 (100%)	11 (100%)
FokI			
Ff	24 (30.1%)	20 (29.4%)	4 (36.4%)
Ff	37 (47.1%)	31 (45.6%)	6 (54.5%)
FF	18 (22.8%)	17 (25%)	1 (9.1%)
Total	79 (100%)	68 (100%)	11 (100%)

The different FokI, but not the BsmI polymorphisms, were significantly associated with reduced BMD. The homozygous presence of the FokI polymorphism (ff) was associated with reduced axial BMD compared with its complete absence (FF) (*p* = 0.022) (Table 5).

**Table 5:** Group differences BMD/ VDR gene polymorphisms (MW ± SD)

	BsmI			
	bb	Bb	BB	*p*-value
FN BMD	0.86 ± 0.12	0.89 ± 0.14	0.85 ± 0.12	n.s
FN T-score	− 1.37 ± 0.94	− 1.04 ± 1.15	− 1.32 ± 0.94	n.s
FN Z-score	− 0.37 ± 0.77	− 0.13 ± 1.06	− 0.35 ± 0.86	n.s
axial BMD	0.89 ± 0.15	1.02 ± 0.18	0.95 ± 0.14	n.s
axial T-score	− 2.16 ± 1.01	− 1.32 ± 1.47	− 1.96 ± 1.20	n.s
axial Z-score	− 1.04 ± 0.69	− 0.26 ± 1.38	− 0.82 ± 1.22	n.s

	FokI			
	ff	Ff	FF	*p*-value
FN BMD	0.83 ± 0.09	0.88 ± 0.13	0.90 ± 0.15	n.s
FN T-score	− 1.43 ± 0.79	− 1.13 ± 1.10	− 1.09 ± 1.16	n.s
FN Z-score	− 0.33 ± 0.70	− 0.33 ± 1.03	− 0.05 ± 0.97	n.s
axial BMD	0.92 ± 0.12	0.99 ± 0.18	0.99 ± 0.18	n.s
axial T-score	− 2.12 ± 0.97	− 1.61 ± 1.47	− 1.46 ± 1.37	0.041*
axial Z-score	− 0.81 ± 0.69	− 0.66 ± 1.48	− 0.30 ± 1.18	n.s

*VDR* vitamin D receptor, *FN* femoral neck, *BMD* bone mineral density, *axial* lumbar spine, *MW* mean, *SD* standard deviation, *n.s.* non-significant

*Post-hoc test for multiple comparisons (Dunn-Bonferroni) ff vs. FF: *p* = 0.021

### Laboratory parameters

Serum calcium levels were slightly elevated in 11 patients and slightly decreased in one patient, while 29 patients (36.7%) showed hypovitaminosis D of varying degrees. 7 patients (9%) showed severe deficiency (< 10 µg/l). These patients did not show statistically significant differences in BMD compared to SSc patients with normal vitamin D levels.

In the present study, 16 SSc patients (20.3%) showed moderate (0.6–2.0 mg/dl) and 6 patients (7.6%) showed a significant CRP elevation (> 2.0 mg/dl). 32 patients (40.5%) showed accelerated ESR. There was no statistical correlation between BMD and inflammatory parameters.

Decreased OC values were found in 3 patients, while 6 patients showed decreased CTX. Elevated OC or CTX values were not present, so no patient was suspected to have increased bone turnover (so-called “high-turnover”).

3 patients had hyperthyroidism, 4 hypothyroidism. 11 patients had elevated serum creatinine.

The different FokI, but not the BsmI genotypes, were in part significantly associated with altered laboratory values. The homozygous presence of a FokI genotype was associated with an elevated ESR compared with its partial or complete absence (*p* = 0.024).

Regarding bone metabolism, CTX values were significantly elevated in patients with homozygous presence of the FokI cleavage side compared to patients with homozygous absence (*p* = 0.014). However, CTX values were all within the reference range regardless of the FokI polymorphism.

The homozygous presence of the FokI cleavage site compared to the homozygous absence was not statistically significant in terms of OC values (*p* = 0.091).

However, CTX and OC values were all within the reference range regardless of the FokI polymorphism (Table 6).

**Table 6:** Group differences VDR gene polymorphisms (BsmI; FokI)/laboratory values (MW ± SD)

	BsmI			
	bb	Bb	BB	p-value
TSH (Ref.: 0.23–4.0 µU/ml)	1.85 ± 1.38	2.09 ± 2.91	1.49 ± 0.88	n.s
Creatinine (Ref.: 0.51–0.95 mg/dl)	0.76 ± 0.17	0.79 ± 0.56	0.76 ± 0.16	n.s
Calcium (Ref.: 2.01–2.55 mmol/l)	2.34 ± 0.1	2.39 ± 0.16	2.43 ± 0.13	n.s
25(OH) Vit. D (Ref.: > 20 µg/l)	32.92 ± 23.39	27.45 ± 16.49	36.16 ± 32.73	n.s
CRP (Ref.: < 0.5 mg/ dl)	1.28 ± 3.41	1.03 ± 2.22	0.4 ± 0.52	n.s
ESR (Ref.: < 20 mm/ h.)	21.22 ± 16.99	23.3 ± 20.08	15.62 ± 10.34	n.s
OC (Ref.: 5.0–55.8 ng/ml)	17.55 ± 9.75	21.15 ± 9.71	19.14 ± 6.9	n.s
CTX (Ref.: 0.1–10.0 ng/ml)	0.32 ± 0.19	0.33 ± 0.23	0.26 ± 0.13	n.s

	FokI			
	ff	Ff	FF	p-value
TSH (Ref.: 0.23–4.0 µU/ml)	1.52 ± 1.06	2.15 ± 3.03	1.8 ± 1.09	n.s
Creatinine (Ref.: 0.51–0.95 mg/dl)	0.89 ± 0.66	0.74 ± 0.23	0.67 ± 0.24	n.s
Calcium (Ref.: 2.01–2.55 mmol/l)	2.41 ± 0.12	2.35 ± 0.14	2.43 ± 0.16	n.s
25(OH) Vit. D (Ref.: > 20 µg/l)	31.37 ± 23.99	30.65 ± 25.75	31.27 ± 17.74	n.s
CRP (Ref.: < 0.5 mg/ dl)	0.72 ± 1.23	0.61 ± 1.27	1.82 ± 4.14	n.s
ESR (Ref.: < 20 mm/ h.)	26.04 ± 14.61	16.92 ± 13.49	21.72 ± 25.32	0.024*
OC (Ref.: 5.0–55.8 ng/ml)	20.26 ± 9.5	21.64 ± 8.85	15.38 ± 7.83	n.s
CTX (Ref.: 0.1–10.0 ng/ml)	0.36 ± 0.23	0.33 ± 0.19	0.21 ± 0.14	0.023*

*TSH* thyroid-stimulating hormone, *25(OH) vit. D* 25-OH vitamin D, *CRP* C-reactive protein, *ESR* erythrocyte sedimentation rate, *OC* osteocalcin, *CTX* β-crosslaps, *Ref* reference range, *MW* mean, *SD* standard deviation, *n.s.* non-significant

*Post-hoc test for multiple comparisons (Dunn-Bonferroni): ESR: FF vs. ff: *p* = 0.044; CTX: FF vs. ff: *p* = 0.014

There was no statistical correlation between the laboratory values and the SSc form.

## Discussion

To our best knowledge, the present study was the first to investigate effects of VDR polymorphisms on BMD in SSc patients, a disease that is overly high associated with osteoporosis.

The data on VDR polymorphisms and BMD were inconclusive for a long period of time. However, in recent years VDR polymorphisms were found and proven to influence BMD negatively. A meta-analysis comprising 16 publications showed that VDR polymorphisms as a genetic factor influences BMD [27]. Several studies of premenopausal and postmenopausal women from different regions of the world showed associations of VDR polymorphisms with the occurrence of osteoporosis [28, 29]. An association between VDR polymorphisms and an increased prevalence of osteoporosis has also been demonstrated in inflammatory rheumatic joint diseases such as RA and AS [16, 21, 22]. In summary, VDR polymorphisms seems to promote BMD reduction up to osteoporosis as a hereditary factor.

In this study, the VDR polymorphisms BsmI and FokI were found in over 70 percent of patients with lc as well as dcSSc. Comparable results were found in Caucasian healthy individuals, as well as RA and axSpA patients [20–22]. So far, only two studies investigated VDR polymorphisms in the context of SSc. However, both studies focused on the relationship between VDR polymorphisms and the likelihood/susceptibility for SSc [30, 31]. In one study there were no statistically significant disparities observed in the allelic and genotype frequencies of ApaI and TaqI polymorphisms between Iranian individuals diagnosed with SSc and a healthy control group [30]. In a Chinese cohort, a statistically significant difference was observed in the distribution of VDR gene polymorphisms among patients compared to a healthy control group for ApaI (98% vs 92%) and BglI (55.6% vs 41%). The distribution of FokI (72.7% vs 76%) and BsmI (100% for both) did not differ significantly. The authors concluded a potential role of VDR polymorphisms in the pathogenesis of SSc[30, 31]. The prevalence of FokI polymorphism found in this study for patients with SSc is comparable with that described in the literature [30].

For the first time, a statistically significant association between BMD and a VDR polymorphism (FokI) could be found in patients with SSc. Patients with SSc who are homozygous carriers of the FokI genotype (ff) are particularly affected by low axial BMD as a significant association between T-score and FokI genotype could be found. Furthermore, there was a numerical reduction in peripheral BMD (measured in both T-score and absolute values in g/cm^2^) which did not reach statistical significance. Even though this study only demonstrated a significant reduction in axial BMD in the context of the FokI polymorphism, both locations are independently important in terms of fracture risk and treatment indication [32, 33]. In addition, FokI genotype was statistic significantly associated with CTX (a bone resorption marker), although changes remained within reference limits.

Our results are in line with previous work in rheumatologic cohorts. Comparative results could be found in patients with AS showing a reduction of axial BMD in male patients [16]. In other studies on VDR polymorphisms in patients with AS, FokI genotype was associated with axial and peripheral BMD reduction [34]. In patients with RA, no differences in BMD were observed in alterations in the FokI polymorphism. However, a statistically significant lower BMD was observed in the presence of a TaqI polymorphism, but only in female patients [21, 22].

For the BsmI genotype polymorphism, no statistical association could be detected regarding a reduction in BMD in the present study. In the comparison of patients with both VDR gene polymorphisms (BsmI and FokI) to patients without both VDR gene polymorphisms, there was no statistically significant difference in axial BMD. An increase in risk due to the presence of multiple VDR gene polymorphisms could therefore not be demonstrated. It should be noted that the low number of patients without both VDR gene polymorphisms compared to those with both polymorphisms (4 vs. 44 patients) greatly limits the statistical power of the analysis.

Moreover, VDR polymorphism was not associated with SSc subtype or gender.

Patients with SSc seem to be a much more vulnerable cohort concerning BMD loss than the normal population. This holds true as 83.5% of patients with SSc showed a reduced BMD (51.9% osteopenia, 31.6% osteoporosis) in our study, which is consistent with other studies [9, 10]. In contrast, a survey on the prevalence of osteoporosis in the female general population in Germany in the age group of 65–74 years showed an osteoporosis prevalence of 17.1% [35]. It should be noted that, unlike in the present study, the survey was limited to women, who generally have a higher risk of osteoporosis compared to men. In the latest survey on the prevalence of osteoporosis in Europe, a prevalence of 6.6% was found for men over 50 years, 22.1% for women over 50 years, and 5.6% for the total population [36]. This study found that VDR gene polymorphisms, especially Fokl polymorphisms, may negatively impact BMD in patients with SSc. Additionally, factors such as early menopause, glucocorticoid treatment, malnutrition, maldigestion, malabsorption, sarcopenia, and decreased mobility likely also play a role in BMD loss, both in general and especially in patients with SSc. [37, 38]. In order to minimize interference and statistical bias, patients with secondary forms of osteoporosis as well as those receiving glucocorticoid therapy were excluded from this study.

In recognition of the enormous prevalence of BMD loss in SSc, it seems reasonable to perform basic osteological diagnostics including bone mineral densitometry in SSc patients on a regular basis. Furthermore, the disease entity SSc is not yet listed as a risk factor for osteoporosis by the umbrella association osteology (DVO), the English osteoporosis guideline, the International Osteoporosis Foundation (IOF) or the FRAX score [39–41]. This should be evaluated critically.

Several limitations of this study need to be addressed. Although a prevalence of VDR polymorphisms could be established, findings are to be interpreted cautiously and seen as preliminary due to the limited number of probands. Further study should evaluate the prevalence further in a multicenter setting to recruit a larger number of probands (as SSc is a rare disease [2]) and to address different ethnic backgrounds. In this regard, seen association of VDR polymorphisms and BMD values should be confirmed in a larger setting. FokI polymorphisms were associated with low axial BMD, however there was numerical change also for peripheral BMD values. A greater number of investigated probands could reveal a more certain association and effect size. Further studies should also consider to recruit a comparator arm, either healthy or as the better option with a different rheumatic disease that is also associated with lower BMD like RA or AS to evaluate not only association but causality. Further studies should evaluate BMD at different time points, as it would be very interesting to evaluate effects of VDR polymorphism on BMD over time.

In conclusion VDR polymorphisms can be frequently found in patients with SSc in comparable prevalence to healthy and rheumatic populations. The homozygous presence of FokI polymorphism, but not BsmI polymorphism, was significantly associated with reduced axial BMD. This could be a possible contributor for the high prevalence of reduced BMD in 83.5% of patients with SSc in this study.

In the era of personalized medicine, a possible implication for clinical practice could be the discussion of the genetic risk of potential osteoporosis based on the detection of VDR polymorphisms, especially in an osteovulnerable population such as patients with SSc.

## Data Availability

The data will be made available on reasonable request.
